# Gender-Specific Association of Oxidative Stress and Inflammation with Cardiovascular Risk Factors in Arab Population

**DOI:** 10.1155/2015/512603

**Published:** 2015-03-31

**Authors:** Abdelkrim Khadir, Ali Tiss, Sina Kavalakatt, Kazem Behbehani, Mohammed Dehbi, Naser Elkum

**Affiliations:** ^1^Department of Biomedical Research, Dasman Diabetes Institute, 15462 Kuwait City, Kuwait; ^2^Diabetes Research Centre, Qatar Biomedical Research Institute, Doha, Qatar; ^3^Clinical Epidemiology Division, Sidra Medical and Research Center, Doha, Qatar

## Abstract

*Background*. The impact of gender difference on the association between metabolic stress and cardiovascular disease (CVD) remains unclear. We have investigated, for the first time, the gender effect on the oxidative and inflammatory stress responses and assessed their correlation with classical cardiometabolites in Arab population. *Methods*. A total of 378 adult Arab participants (193 females) were enrolled in this cross-sectional study. Plasma levels of CRP, IL-6, IL-8, TNF-*α*, ROS, TBARs, and PON1 were measured and correlated with anthropometric and cardiometabolite parameters of the study population. *Results*. Compared to females, males had significantly higher FBG, HbA1c, TG, and blood pressure but lower BMI, TC, and HDL (*P* < 0.05). After adjustment for BMI and WC, females had higher levels of ROS, TBARS, and CRP (*P* < 0.001) whereas males had increased levels of IL-8, IL-6, and TNF-*α* (*P* < 0.05). Moreover, after adjustment for age, BMI, and gender, the levels of TNF-*α*, IL-6, and ROS were associated with central obesity but not general obesity. *Conclusion*. Inflammation and oxidative stress contribution to CVD risk in Arab population linked to gender and this risk is better reflected by central obesity. Arab females might be at risk of CVD complications due to increased oxidative stress.

## 1. Introduction

Obesity is a worldwide epidemic with a major burden on the healthcare systems and is now recognized as a disease (http://www.ama-assn.org/). Obesity is an important determinant of cardiovascular diseases (CVD) as it promotes a cluster of CVD risk factors including dyslipidemia, type-2 diabetes (T2D), and hypertension [1]. The Arab population of the Gulf Cooperation Council (GCC) countries has experienced rapid socioeconomic growth and changes over the last two decades, resulting in a tremendous increase in obesity as well as its associated comorbidities [2]. Indeed, GCC countries including Kuwait are ranked among top 10 countries with the highest prevalence of T2D worldwide [3]. Kuwait has also the highest rate of obesity in the region with more than 45% of adults being obese: 53% in females as compared to 39% in males [4].

Chronic inflammation and excessive oxidative stress are key pathophysiological hallmarks of obesity and their contribution to downstream complications is well established [5, 6]. Sedentary lifestyle, physical inactivity, and overconsumption of a calorie-rich diet are among the modifiable factors that lead to this chronic condition [7]. White adipose tissue has been identified as the predominant site that produces various bioactive molecules including cytokines, adipokines, CRP, and free fatty acids which lead to aberrant regulation of inflammatory and stress kinases and ultimately a loss of cellular homeostasis [8].

Although chronic inflammatory response is an established fact in obesity, the molecular determinants that trigger this response and maintain it in a sustained state are still poorly understood. Reactive oxygen species (ROS) and free fatty acids have however been proposed as potential contributors to this process [9]. Indeed, conditions that lead to increased oxidative stress are also known for their ability to lead to inflammation, in large part through the activation of NF-*κ*B [10]. In turn, activated inflammatory cells release high levels of ROS that potentiate the inflammatory response [6]. Thus, the relationship between oxidative stress and inflammation is more complex than it was originally thought, and it is clear that inflammation and oxidative stress are mutually inclusive and most likely they operate by creating a cycle which exacerbates them [6].

It is well established that oxidative stress is caused by an imbalance between increased production of ROS and reduced antioxidant defense. For instance, paraoxonase-1 (PON1) is an antioxidative enzyme preventing the formation of oxidized lipoproteins [11]. PON1 is synthesized primarily in the liver and is secreted into the bloodstream, where it protects phospholipids by associating with high-density lipoproteins (HDL) [12].

The status of oxidative stress and inflammatory response with respect to gender effect on CVD risk factors has been investigated in different ethnic populations and clear gender-related effects were reported [13–15]. These dissimilarities in plasma levels are often attributed to physical and physiological differences between males and females, such as adiposity distribution, lean muscle mass, and differences in hormone levels [13–15]. Therefore, expanding these studies by further stratifying various ethnic groups according to gender will provide insights regarding the gender effect on the relationship between inflammatory and stress markers and their association with CVD risk factors.

As studies that investigated these two responses in the Arab population are scarce, we designed the present study as a cross-sectional analysis on 378 Arab adults living in Kuwait in order to assess gender differences in oxidative stress and inflammatory circulating markers and their association with cardiometabolites. This study is of emerging interest in CVD research, since the use of circulating biomarkers might help in the stratification of CVD risk factors.

## 2. Materials and Methods

### 2.1. Study Participants

This population-based cross-sectional survey was carried out between June 2011 and August 2012 and was undertaken on adult male and female Arab emigrants in Kuwait. According to the 2011 Kuwait consensus, 67.7% of the population in Kuwait were expatriates hailing mostly from Arab countries such as Egypt, Syria, Lebanon, Palestine, Jordan, and Arab Gulf countries. Subjects were selected randomly from the computerized register of the Public Authority of Civil Information. The study conformed to the principles outlined in the Declaration of Helsinki and was approved by the institute's Scientific Advisory Board and Ethical Review Committee at Dasman Diabetes Institute (DDI). An informed written consent was obtained from all the participants before their enrolment in the study.

### 2.2. Anthropometric and Physical Measurements

Physical and anthropometric measurements included body weight, height, waist circumference (WC), and blood pressure (BP). BP was measured with Omron HEM-907XL digital sphygmomanometer and an average of 3 readings, with 5–10-minute rest between each, was noted. Height and weight were measured with participants wearing light indoor clothing and bare-footed using calibrated portable electronic weighing scales and portable inflexible height measuring bars. WC was measured using constant tension tape at the end of a normal expiration, with arms relaxed at the sides, at the highest point of the iliac crest at the midaxillary line. Body mass index (BMI) was calculated, using standard metric BMI formula (Kg/m^2^). Obesity was assessed using BMI cut-off standard criteria; BMI between 18.5 and 24.9 was considered normal, 25 to 29.9 was considered overweight, and equal to or higher than 30 was considered obese. According to international diabetes federation (IDF), abdominal obesity (central obesity) was defined as WC ≥94 cm in males and ≥80 cm in females.

### 2.3. Laboratory Measurements

#### 2.3.1. Blood Samples

Blood samples were obtained after an overnight fast of at least 10 hours and analyzed for fasting blood glucose (FBG), HbA1c, fasting insulin, and a lipid profile that included triglycerides (TG), total cholesterol (TC), low-density lipoprotein (LDL), and high-density lipoprotein (HDL). All laboratory tests were performed by certified technicians at the clinical laboratories of DDI using the Ministry of Health approved methods and quality standards. Insulin resistance was calculated using Homeostasis Model Assessment (HOMA-IR) formula: fasting insulin (mU/L) × FBG (mmol/L)/22.5 [16]. To measure inflammatory and metabolic biomarkers, blood was drawn into EDTA tubes. Plasma was obtained after centrifugation at 1700 ×g for 15 min at 4°C, aliquoted, and then stored at −80°C until assayed.

#### 2.3.2. Analytical Measurements

Plasma levels of inflammatory markers were assessed with the multiplexing immunobead array using the Bio-Plex Pro human Cytokine 27-Plex immunoassay kit that included IL-6, IL-8, and TNF-*α* (Bio-Rad, Hercules, CA). Data was analyzed with Bio-Plex manager software version 6 (Bio-Rad, Hercules, CA). Lipid peroxidation was assessed by measuring plasma levels of malonaldehyde, using TBARs Assay Kit (Cayman Chemical Company, Ann Arbor, MI). Serum levels of ROS were determined using the OxiSelect ROS Assay Kit (Cell Biolabs Inc., San Diego, CA). Paraoxonase-1 activity was measured using EnzChek Paraoxonase Assay Kit (Life Technologies, Grand Island, NY). C-reactive protein (CRP) levels were measured using high sensitivity CRP “hsCRP” ELISA kit (Biovendor, Asheville, NC).

All the abovementioned assays were carried out according to the instructions of the manufacturers.

### 2.4. Statistical Analysis

All analyses were performed using SAS (version 9.4; SAS Institute, Cary, NC). Normality tests were run to assess data distribution. Comparisons of subjects characteristics between genders were made by Student's *t*-test or Wilcoxon test for nonparametric analyses in variables with nonnormal distribution. Spearman correlation coefficients were estimated to determine associations between levels of the various inflammatory and oxidative stress markers investigated and anthropometric and clinical measurements. Age, BMI, and WC/gender adjusted geometric mean values of various inflammatory and oxidative stress markers concentrations in men and women were calculated using analysis of covariance (ANCOVA). To examine independent correlates of various inflammatory and oxidative stress markers, multivariate linear regression models were constructed with log-transformed biomarkers as the dependent variable. All data were reported as mean ± standard deviation (SD) and range, unless otherwise stated. Research Electronic Data Capture (REDCap) was used for data collections and data management [17]. All statistical assessments were two-sided and considered significant at *P* value < 0.05.

## 3. Results

### 3.1. Characteristics of the Study Population

The anthropometric and clinical parameters of the study population are shown in Table 1. A total of 378 participants consisting of 185 males and 193 females matched for age were enrolled in this study with a mean age of 44.4 ± 11.7 years. Females had higher BMI but lower WC than males (*P* < 0.0001 and *P* = 0.0153, resp.). By contrast, the SBP and DBP were significantly higher in males compared to females (*P* < 0.0001 and *P* = 0.0252, resp.). Likewise, the glycemic markers were higher in males than in females as monitored by FBG (*P* = 0.0424) and HbA1c (*P* < 0.0001). There was also an alteration of lipid profile between the two groups as females had increased levels of both cholesterol (*P* = 0.0353) and HDL (*P* < 0.0001) but decreased levels of TG (*P* = 0.0045). In our study population, 206 subjects showed high blood pressure, including 105 subjects previously diagnosed, of which only 77 were taking medication. Regarding diabetes, 109 were diabetic; among them 98 subjects were previously diagnosed and 92 receiving treatment. 27 subjects (7.2%) already had a history of CVD; however, for our study, we only collected data related to diabetes treatment. According to our survey, females were more physically active and smoked less than males (*P* = 0.0026 and *P* < 0.0001, resp.).

### 3.2. Status of the Inflammatory and Oxidative Stress Responses in the Studied Arab Population

**Figure 1:**
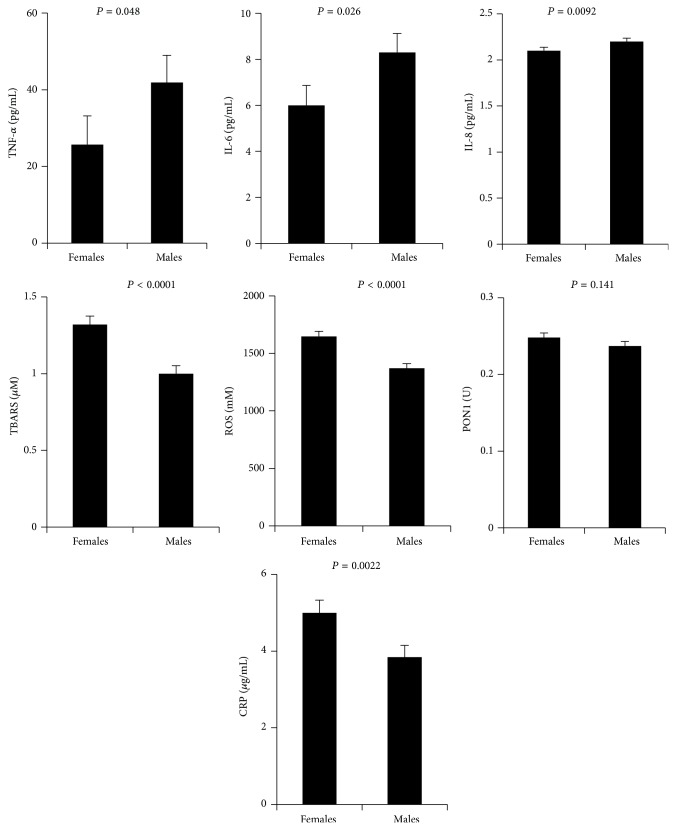
Least square means of TNF-*α*, Il-6, IL-8, CRP, TBARs, ROS, and PON1 concentrations in plasma, for all subjects after adjusting for age, BMI, and WC.

Gender differences in circulating makers of inflammation (CRP, IL-6, IL-8, and TNF-*α*) and oxidative stress (ROS, TBARS, and PON1) were measured and the medians are displayed in Table 2. Accordingly, the levels of CRP, ROS, and TBARS were all significantly high in females compared to males (*P* < 0.0001). By contrast, males had higher levels of IL-8 (*P* = 0.0032) and the levels of IL-6, TNF-*α*, and PON1 were comparable between the two genders (Table 2). After adjustment for age, BMI, and WC, similar patterns between males and females were observed for CRP (*P* = 0.0022), IL-8 (*P* = 0.0092), TBARS (*P* < 0.0001), and ROS (*P* < 0.0001) (Figure 1). While there was still no significant difference between genders in the activity of PON1, the concentration levels of IL-6 and TNF-*α* were significantly higher in males than females (*P* < 0.05).

### 3.3. Correlation of Inflammatory and Oxidative Stress Markers with Anthropometric and Clinical Parameters

These observations prompted us to investigate the possible associations between these markers and various anthropometric and clinical parameters of our study population. To this end, we performed an initial Spearman correlation on all subjects and the data are displayed in Table 3. As shown, CRP correlated positively with most of the parameters but the highest significant correlations were found with BMI, WC, and HOMA-IR (*P* < 0.0001). Other positive correlations were also found for TC and LDL (*P* < 0.001), TG (*P* < 0.01), and DBP and HbA1c (*P* < 0.05). SBP and HDL did not correlate with CRP (Table 3). Similar to CRP, IL-6 correlated positively with age and BMI (*P* < 0.01), WC (*P* < 0.001), and FBG, HbA1c, and HOMA-IR (*P* < 0.05) but negatively with HDL (*P* < 0.05). In the case of IL-8, a positive correlation was found for age, HbA1c, and TG (*P* < 0.001) as well as WC, HOMA-IR, and TC (*P* < 0.05) but a negative correlation with HDL (*P* < 0.05) (Table 3). By contrast to the inflammatory markers, we observed less correlations with the oxidative stress markers but, as shown in Table 3, a negative correlation was observed between TBARS and BMI (*P* < 0.05) and a positive correlation between ROS and HbA1c (*P* < 0.0001). To investigate the specific gender effect of each of these markers with the anthropometric and clinical parameters, we performed a gender-based Spearman correlation and the results are displayed in Table 4. Under our conditions, CRP levels in females were associated positively with TC (*P* < 0.001). In males, however, CRP correlated positively with SBP and negatively with HDL (*P* < 0.001). TNF-*α* correlated positively with TC in females (*P* < 0.05). IL-6 correlated in females positively with FBG, HOMA-IR, TC, and TG (*P* < 0.05). IL-8 correlated also in females positively with HOMA-IR, TC, and TG (*P* < 0.05) but negatively with HDL (*P* < 0.05) (Table 4). For the markers of oxidative stress, TBARS levels correlated positively in females with HDL (*P* < 0.05), whereas PON1 levels correlated negatively with FBG, HbA1c, and TG (*P* < 0.05) and positively with HDL (*P* < 0.05). In males, PON1 levels correlated positively with TC (*P* < 0.01) and LDL (*P* < 0.05) (Table 4).

### 3.4. Association Analysis of Central Obesity with the Inflammatory and Oxidative Stress Markers

**Figure 2:**
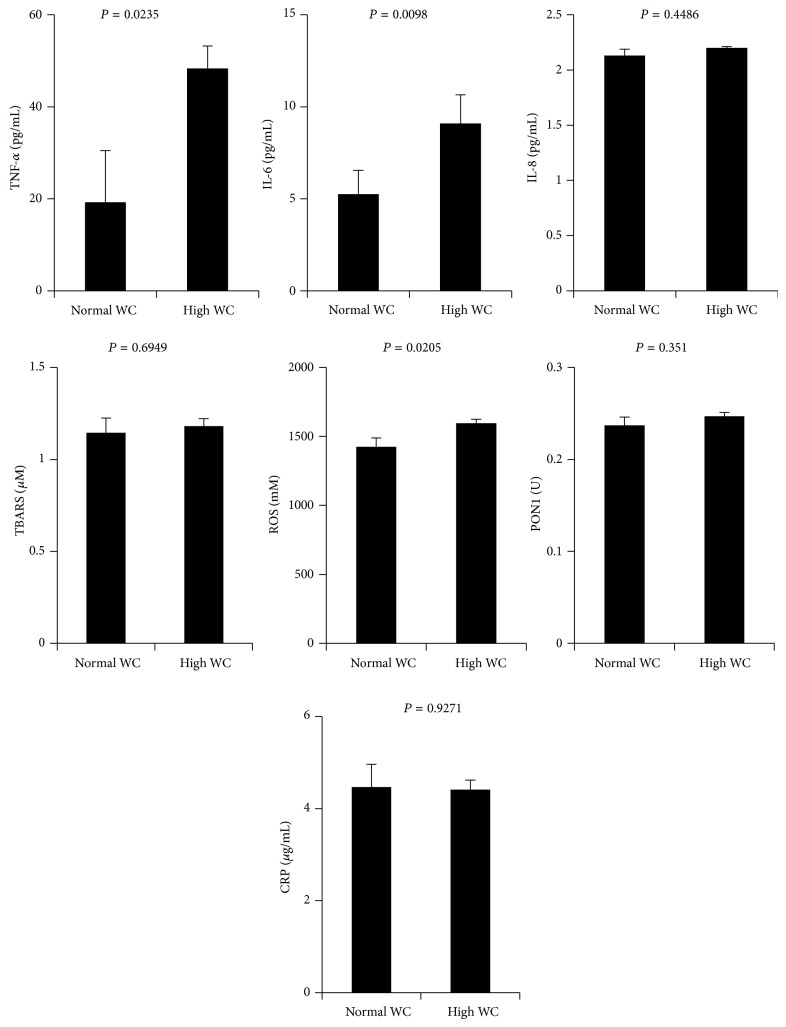
Least square of TNF-*α*, Il-6, IL-8, CRP, TBARs, ROS, and PON1 concentrations in plasma, for all subjects after adjusting for age, BMI, and gender.

We next investigated the possible association of central obesity with the inflammatory and oxidative stress markers after adjusting for age, gender, and BMI. To this end, we choose two groups within our study population with either normal or higher central obesity as measured by WC and the data are illustrated in Figure 2. As shown, our data indicated that, out of the seven markers investigated, central obesity was associated with increased levels of TNF-*α* (*P* < 0.05), IL-6 (*P* < 0.01), and ROS (*P* < 0.05).

## 4. Discussion

The increase in the incidence and prevalence of obesity and its related comorbidities in the countries of the GCC are of extreme health and socioeconomic concerns. Nonnational Arabs living in the GCC region are also vulnerable to these chronic disorders, highlighting the pivotal role of environmental factors, namely, excessive food energy intake, sedentary lifestyle, and physical inactivity, as major contributors to these chronic conditions. In this study, we investigated the status of the inflammatory and oxidative stress responses in nonnational Arabs of Kuwait with and without central obesity and evaluated whether there was a gender difference in these two responses as well as their association with classical CVD risk factors. After adjustment for BMI and WC, the main findings of the study are as follows: (1) higher levels of TNF-*α*, IL-6, and IL-8 were observed in males, whereas females had higher levels of CRP, ROS, and TBARS; (2) the levels of TNF-*α*, IL-6, and ROS were associated more with WC rather than the BMI; (3) despite their apparent healthier status, Arab females might be at risk of CVD complication due to increased oxidative stress. These results support the hypothesis that central adiposity dysfunction may play a critical role in the cross talk between biomarkers of inflammation and oxidative stress and probably other metabolites to constitute major CVD risk factors that may indirectly contribute to CVD.

To the best of our knowledge, this was the first cross-sectional study that investigated the status of the inflammatory and oxidative stress responses in nonnational Arabs of Kuwait with relationship to central obesity and the risk of CVD. Although direct causality cannot be inferred from these correlation studies, our data provide an additional support that warrants further investigations to understand the cross talk between inflammation and oxidative stress with respect to gender and their pathophysiological impacts on metabolic diseases.

The status of the inflammatory and oxidative stress responses with respect to obesity and its association with CVD risk factors has been investigated intensively in several cross-sectional studies involving many ethnic groups in both males and females [18–25]. These studies were pivotal in pointing to the existence of gender differences in the inflammatory and stress responses. More importantly, they revealed the existence of a myriad of factors including the proportion of fat tissue and its distribution, the level of sex hormones, genetic factors, lifestyle habits that could contribute to these differences, and ultimately the gender-specific risk of metabolic diseases. Consistent with these studies, the levels of circulating TNF-*α*, IL-6, and ROS in our study population were associated with central obesity after adjusting for age, gender, and BMI (Figure 2). The observed profile appeared to be specific to WC as there was no significant association between these markers and the BMI of our study population when adjusted for age, gender, and WC (data not shown). Our findings corroborate and further extend those obtained from the Framingham heart study in which indicators of central adiposity and not global obesity were strongly associated with inflammation and oxidative stress markers [22]. In agreement with these findings, a more recent study carried out on 100 Emirati obese-diabetic subjects consisting of 41 males and 59 females reported also that WC indicated adiposity better than BMI and that increased WC associated with increased inflammation and oxidative stress [24]. They also reported that females had higher CRP and decreased antioxidant status (vitamin C) and HDL and that these levels were significantly associated with adiposity as measured by WC [24]. Likewise, a more closely related study that investigated the gender differences in fat distribution and inflammation in Arabs was carried out on 58 healthy Qatari consisting of 29 males and 29 females [20]. In their study, while there was no significant difference in the levels of CRP and IL-6 between genders, they found a strong correlation of CRP and IL-6 with WC and SBP in males. The above findings did not fully agree with another investigation that was carried out recently on 1005 relatively lean Chinese females in which both WC and BMI were associated with various markers of inflammation, namely, CRP, TNF-*α*, soluble TNF-receptor 1 (sTNF-R1), and IL-6, and the marker of oxidative stress F2-IsoP-M [19]. Other studies have also shown that female gender and BMI are independent risk factors of metabolic syndrome in Turkish and Gaza Strip populations [26, 27]. Relationships between anthropometric adiposity indicators and CVD risk factors have been already thoroughly explored in many ethnic groups. Our Spearman correlation analysis showed that inflammatory markers (IL-6, IL-8, and CRP) correlated positively with adiposity (WC and BMI) and with the classical cardiometabolites (blood pressure, sugar index, and lipids index) in whole population (Table 3). However, after adjusting our data for WC, correlation with BMI became statistically insignificant (data not shown). Indeed, the majority of studies suggest that WC is a better indicator of CVD risk than BMI and has been proven to be an appropriate clinical measure of the pathogenic potential of adipose tissue amongst populations [28–30].

In our current investigation, we adjusted for age, BMI, and WC and found that CRP, ROS, and TBARS levels were significantly higher in females whereas males had increased levels of TNF-*α*, IL-6, and IL-8 (Figure 1). Consistent with these findings, Cartier et al. reported also increased levels of CRP in premenopausal females and increased levels of IL-6 and TNF-*α* in males [18]. Other independent studies have also demonstrated higher levels of oxidative stress markers in females as compared to males [31, 32] and it was suggested that oxidative stress may be a key contributor to increased CVD risk in females, presumably through CRP [30, 31]. CRP was initially described as an inflammatory marker of CVD [33]; however, recent evidence indicated its link to oxidative stress [34, 35]. Moreover, Cartier et al. concluded from their study that the observed gender difference in CRP concentrations cannot be explained by the visceral adipose tissue depots. Instead, they suggest that the higher CRP concentrations found in women are linked to higher subcutaneous fat in women [15]. In agreement with these hypotheses, our results showed that females display higher CRP levels after correction for adiposity markers as compared to males and when adjusted for gender and BMI, CRP did not associate with WC (Figure 2).

The high BMI and WC as well as elevated TC and LDL in females enrolled in our study may explain this increase in oxidative stress response as compared to males. It is generally accepted that the prevalence of CVD is higher in males and postmenopausal females [36, 37]. However, epidemiological data carried out on Arab population indicated that CVD associated risks are elevated even in younger females compared to other ethnic groups [38, 39]. The current study sheds light on those risks in premenopausal females and may suggest that impairment of the oxidative stress response may be a key CVD risk factor rather than classical risk factors. In support of this, lower levels of glucose levels, dyslipidemia, and hypertension were observed in females as compared to males (Table 1). By contrast, males displayed lower oxidative stress despite their high BMI, WC, FBG, TG, and LDL (Table 2).

Classifying the study subjects into different categories of obesity might reflect better the link of adiposity distribution on inflammation and oxidative stress. Indeed, obesity is described as being a state of low-grade chronic inflammation in which increased IL-6, IL-8, and TNF-*α* levels have been described as obesity-induced risk factor of CVD [40, 41]. Adipose tissue is a potent source of proinflammatory and redox mediators, suggesting that sex differences in total and regional adiposity could influence risk of CVD. Evidence showed that lipid ratio, in particular TG/HDL, performs better than individual lipids in predicting CVD risk [42]. Our data showed that this ratio is higher in males than in females (data not shown). Moreover, lipid ratios displayed a strong correlation with inflammation (IL-6, IL-8, and TNF-*α*), particularly the TG/HDL and its logarithmic function (AIP), which also associated negatively with the antioxidant enzyme PON1 in males (data not shown). The latter associations could reflect a direct effect of systemic inflammatory stress on lipoproteins or indirectly through metabolic alterations. Accordingly, recent study reported that dysfunctional HDL might be considered as novel target in CVD [43]. Although the interaction between inflammatory mediators and lipoproteins is not clear, there is increasing evidence that the systemic inflammatory state potentiates CVD such as atherosclerosis [44].

It should also be noted that not all obese individuals are at a similar risk of these complications, since a significant proportion of obese individuals are metabolically healthy [45]. Prevalence of this healthy obese phenotype was reported to be 2 to 4 times higher in females as compared to males [46]. Interestingly, a recent study showed a positive association between metabolically healthy obese phenotypes in females having a moderate to high level of physical activity [47]. They further suggested that metabolically healthy obese individuals may have a reduced inflammatory status [47]. In agreement with this observation, our data indicated that females displayed healthier metabolic profile and lifestyle as compared to males. This suggests that factors other than simple energy imbalance, such as oxidative stress, might determine the obesity risk outcomes. Hence, the increased oxidative stress observed in the current investigation in apparently healthy females supports the concept that oxidative stress could represent a reliable predictor of obesity and CVD in females [48]. Thus, Arab females are probably more prone to the detrimental effects of oxidative stress at lower blood glucose, lipid thresholds, and inflammation than males.

Our study has, however, several limitations that deserve consideration for future follow-up studies. For instance, this is a cross-sectional study from which it is difficult to extract meaningful conclusions regarding direct causality of CVD. Second, given the complex cross talk between the inflammatory and stress responses [49], it is still difficult to identify the triggers that initiate such responses and to establish the hierarchical sequence of events that lead to a perturbation of normal homeostasis. Furthermore, our study used an indirect measurement for generalized adiposity and central adiposity indicators, which probably have less accuracy, compared to direct measurement methods. Furthermore, other confounding factors such as diet, genetic determinants, and sociocultural conditions may affect our findings. Additionally, we used international standards for cut-off points for BMI and WC and this might not be fully accurate for Arabs as was reported previously [50]. Our findings might be improved once appropriate cut-off points for Arabs are established.

## 5. Conclusions

Despite these limitations, our study is the first that investigated the association of classical CVD risk factors with oxidative stress and inflammation in the Arab population living in Kuwait. Having a relatively healthier metabolic profile compared with males, females showed an association with oxidative stress markers which may be considered as a risk factor of CVD regardless of their lower inflammatory status. Males, however, displayed a metabolically unhealthy profile with increased inflammation levels probably reflecting their poor lifestyle including lower physical exercise and higher smoking rates. A further study is currently underway to prospectively follow up this cohort to determine directionality of the observed associations and trends.

## Figures and Tables

**Table 1:** Anthropometric and clinical characteristics of the Arab population study.

Characteristics	All subjects (*n* = 378)	Females (*n* = 193)	Males (*n* = 185)	^∗^ *P* value
Age (years)	44.4 ± 11.7	44.06 ± 11.9	44.78 ± 11.51	0.5109
BMI (kg/m^2^)	31.9 ± 6.4	33.24 ± 6.67	30.48 ± 5.80	**<0.0001**
WC (cm)	101.0 ± 14.3	99.28 ± 14.26	102.7 ± 14.13	**0.0153**
SBP (mmHg)	129.9 ± 19.8	124.8 ± 19.6	135.1 ± 18.5	**<0.0001**
DBP (mmHg)	78.3 ± 12.5	76.89 ± 12.6	79.77 ± 12.3	**0.0252**
FBG (mmol/L)	6.25 ± 3.0	5.93 ± 2.5	6.59 ± 3.5	**0.0424**
HbA1c (%)	6.07 ± 1.8	5.66 ± 1.4	6.53 ± 2.0	**<0.0001**
HOMA-IR	3.30 ± 3.95	3.44 ± 4.7	3.14 ± 2.9	0.4486
TC (mmol/L)	5.19 ± 1.12	5.31 ± 1.08	5.06 ± 0.08	**0.0353**
TG (mmol/L)	1.71 ± 1.19	1.54 ± 0.06	1.89 ± 0.11	**0.0045**
HDL (mmol/L)	1.12 ± 0.35	1.25 ± 0.02	0.99 ± 0.02	**<0.0001**
LDL (mmol/L)	3.35 ± 1.00	3.41 ± 0.07	3.28 ± 0.07	**0.2305**
Hypertension (%)	54.5	43.0	66.5	**<0.0001**
Diabetes (%)	28.3	23.8	32.9	**0.0487**
CVD (%)	7.2	6.3	8.2	0.4481
Current smoking status (%)	45.9	26.9	73.1	**<0.0001**
Physically active (%)	58.8	57.4	42.6	**0.0026**

Data are presented as mean ± SD. BMI: body mass index; WC: waist circumference; SBP: systolic blood pressure; DBP: diastolic blood pressure; FBG: fasting blood glucose; TC: total cholesterol; TG: triglycerides; HDL: high-density lipoprotein; LDL: low-density lipoprotein; CVD: cardiovascular disease. ^∗^ *P* value shows the differences between males and females.

**Table 2:** Circulating levels of the inflammatory and oxidative stress markers in the studied Arab population.

Markers	Females (*n* = 193)	Males (*n* = 185)	*P* value
CRP (*μ*g/mL)	4.53 (0.01–21.9)	2.10 (0.01–18.2)	**<0.0001**
IL-6 (pg/mL)	6.45 (1.7–36.2)	6.60 (1.3–121.5)	0.2851
IL-8 (pg/mL)	7.9 (2.8–177.5)	8.8 (3.5–56.6)	**0.0032**
TNF-*α* (pg/mL)	30.7 (4.6–151.8)	32.5 (1.7–355.4)	0.2461
ROS (mM)	1665.2 (906.4–3071.3)	1424.8 (850.9–2703.1)	**<0.0001**
TBARS (*μ*M)	1.24 (0.29–5.05)	0.94 (0.23–3.41)	**<0.0001**
PON1 (U)	0.25 (0.08–0.56)	0.24 (0.04–0.44)	0.1361

Results are reported as median (range).

**Table 3:** Spearman correlation of inflammatory and oxidative stress markers with cardiometabolic risk factors in all study subjects.

Parameters	CRP	TNF-*α*	IL-6	IL-8	TBARS	ROS	PON1
Age	0.12	0.06	0.18^∗∗^	0.17^∗∗^	0.002	−0.01	−0.09
BMI	0.53^§§^	0.01	0.15^∗∗^	0.10	−0.11^∗^	−0.02	−0.0007
WC	0.43^§§^	0.03	0.18^§^	0.12^∗^	−0.09	0.05	−0.01
SBP	0.09	−0.03	0.05	0.04	−0.01	−0.07	0.07
DBP	0.14^∗∗^	−0.03	−0.02	−0.008	0.01	−0.02	0.03
FBG	0.14^∗∗^	0.07	0.12^∗^	0.10	0.05	0.01	−0.06
HbA1c	0.12^∗^	0.05	0.12^∗^	0.17^∗∗^	0.02	0.22^§§^	−0.05
HOMA-IR	0.36^§§^	0.05	0.14^∗^	0.14^∗^	0.04	0.04	0.03
TC	0.19^§^	0.11^∗^	0.10	0.13^∗^	−0.05	−0.006	0.06
HDL	−0.05	−0.08	−0.14^∗^	−0.13^∗^	−0.004	0.08	0.06
LDL	0.176^§^	0.07	0.07	0.08	−0.08	−0.02	0.07
TG	0.14^∗∗^	0.06	0.08	0.15^∗∗^	0.03	−0.01	−0.03

Values are Spearman correlation coefficients. ^∗^ *P* < 0.05; ^∗∗^ *P* < 0.01; ^§^ *P* < 0.001; ^§§^ *P* < 0.0001.

**Table 4:** Spearman correlation of inflammatory and oxidative stress markers with cardiometabolic risk factors according to gender.

Parameters	CRP		TNF-*α*		IL-6		IL-8		TBARS		ROS		PON1	
	Females	Males	Females	Males	Females	Males	Females	Males	Females	Males	Females	Males	Females	Males
Age	0.13	0.15^∗^	<0.1	<0.1	0.17^∗^	0.14	0.18^∗^	0.14	<0.1	<0.1	−0.12	0.17^∗^	<0.1	−0.17^∗^
BMI	0.58^§§^	0.38^§§^	<0.1	<0.1	0.12	0.14	<0.1	<0.1	<0.1	<0.1	<0.1	<0.1	<0.1	<0.1
WC	0.57^§§^	0.36^§§^	<0.1	<0.1	0.15^∗^	0.20^∗∗^	<0.1	<0.1	<0.1	<0.1	<0.1	0.14	<0.1	<0.1
SBP	0.25^§^	0.12	<0.1	<0.1	<0.1	<0.1	<0.1	<0.1	<0.1	<0.1	<0.1	<0.1	<0.1	<0.1
DBP	0.20^∗∗^	0.18^∗^	<0.1	<0.1	<0.1	<0.1	<0.1	<0.1	<0.1	<0.1	<0.1	<0.1	<0.1	<0.1
FBG	0.21^∗∗^	0.14	<0.1	<0.1	<0.1	0.14^∗^	<0.1	0.12	<0.1	<0.1	<0.1	<0.1	<0.1	−0.19^∗^
HbA1c	0.25^§^	0.16^∗^	<0.1	<0.1	<0.1	0.11	0.12	0.10	<0.1	0.14	0.18^∗^	0.25^∗∗^	0.10	−0.19^∗^
HOMA-IR	0.43^§§^	0.31^§§^	<0.1	<0.1	<0.1	0.18^∗^	<0.1	0.16^∗^	<0.1	<0.1	<0.1	<0.1	<0.1	<0.1
TC	0.10	0.25^§^	<0.1	0.18^∗^	<0.1	0.16^∗^	<0.1	0.18^∗^	<0.1	<0.1	<0.1	<0.1	0.20^∗∗^	<0.1
HDL	−0.26^§^	<0.1	<0.1	<0.1	<0.1	−0.11	<0.1	−0.17^∗^	<0.1	0.15^∗^	<0.1	<0.1	0.12	0.17^∗^
LDL	0.17^∗^	0.17^∗^	<0.1	0.11	<0.1	0.10	<0.1	0.13	<0.1	<0.1	<0.1	<0.1	0.16^∗^	−0.01
TG	0.17^∗^	0.21^§^	<0.1	0.14	<0.1	0.15^∗^	0.13	0.19^∗^	<0.1	<0.1	<0.1	<0.1	<0.1	−0.15^∗^

Values are Spearman correlation coefficients. ^∗^ *P* < 0.05; ^∗∗^ *P* < 0.01; ^§^ *P* < 0.001; ^§§^ *P* < 0.0001.
